# *N*-(4-Methyl­benz­yl)-3-nitro­anilinium chloride

**DOI:** 10.1107/S1600536812026281

**Published:** 2012-06-20

**Authors:** Marijana Đaković, Tomislav Portada, Dora Ugrinovski

**Affiliations:** a Department of Chemistry, Faculty of Science, University of Zagreb, Horvatovac 102a, HR-10000 Zagreb, Croatia; b Department of Organic Chemistry and Biochemistry, Ruder Bošković Institute, PO Box 180, HR-10000 Zagreb, Croatia; c 5^th^ High School, Klaićeva 1, HR-10000 Zagreb, Croatia

## Abstract

The cation of the title compound, C_14_H_15_N_2_O_2_^+^·Cl^−^, comprises two almost ideally planar systems, 3-nitro­phenyl (r.m.s. deviation = 0.0117 Å) and 4-methyl­phenyl (r.m.s. deviation = 0.238 Å), separated by the central C—N bond, and with their mean planes inclined to one another by 61.36 (5)°. In the crystal, hydrogen-bonded chains running along [001] are generated by connecting neighbouring mol­ecules *via* N—H⋯Cl hydrogen bonds and consolidated by C—H⋯Cl and C—H⋯O inter­actions. Within these chains, fused *R*_2_^1^(6) and *R*_3_^2^(10) ring motifs are formed. Parallel chains are further linked into a two-dimensional network parallel to (100) *via* C—H⋯O inter­actions.

## Related literature

For the crystal structure of the free base, *N*-(4-methyl­benz­yl)-3-nitro­aniline, see: Đaković *et al.* (2012 ▶). For the crystal structures of hydro­chloride salts of similar *N*-benzyl­anilines, see: Dai *et al.* (2010 ▶); Albrecht *et al.* (2010 ▶); Boulcina *et al.* (2011 ▶). For graph-set theory, see: Etter (1990 ▶); Bernstein *et al.* (1995 ▶). 
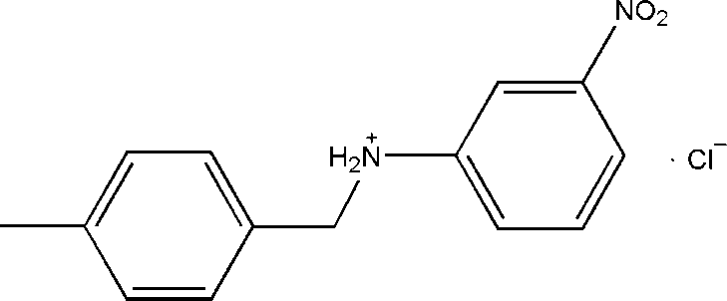


## Experimental

### 

#### Crystal data

C_14_H_15_N_2_O_2_^+^·Cl^−^
*M* *_r_* = 278.73
Monoclinic, 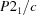

*a* = 14.2586 (7) Å
*b* = 13.1416 (8) Å
*c* = 7.7524 (3) Å
β = 105.215 (5)°
*V* = 1401.73 (13) Å^3^
*Z* = 4
Mo *K*α radiation
μ = 0.27 mm^−1^
*T* = 296 K
0.56 × 0.46 × 0.14 mm

#### Data collection

Oxford Diffraction Xcalibur diffractometer with a Saphire-3 CCD detector
Absorption correction: multi-scan (*CrysAlis PRO*; Oxford Diffraction, 2009 ▶) *T*_min_ = 0.852, *T*_max_ = 0.964
8156 measured reflections
4075 independent reflections
2357 reflections with *I* > 2σ(*I*)
*R*_int_ = 0.024

#### Refinement

*R*[*F* ^2^ > 2σ(*F* ^2^)] = 0.043
*wR*(*F* ^2^) = 0.113
*S* = 0.88
4075 reflections
181 parameters
H atoms treated by a mixture of independent and constrained refinement
Δρ_max_ = 0.31 e Å^−3^
Δρ_min_ = −0.20 e Å^−3^

### 

Data collection: *CrysAlis PRO* (Oxford Diffraction, 2009 ▶); cell refinement: *CrysAlis PRO*; data reduction: *CrysAlis PRO*; program(s) used to solve structure: *SHELXS97* (Sheldrick, 2008 ▶); program(s) used to refine structure: *SHELXL97* (Sheldrick, 2008 ▶); molecular graphics: *ORTEP-3* (Farrugia, 1997 ▶) and *Mercury* (Macrae *et al.*, 2008 ▶); software used to prepare material for publication: *SHELXL97* and *PLATON* (Spek, 2009 ▶).

## Supplementary Material

Crystal structure: contains datablock(s) global, I. DOI: 10.1107/S1600536812026281/su2452sup1.cif

Structure factors: contains datablock(s) I. DOI: 10.1107/S1600536812026281/su2452Isup2.hkl

Supplementary material file. DOI: 10.1107/S1600536812026281/su2452Isup3.cml

Additional supplementary materials:  crystallographic information; 3D view; checkCIF report

## Figures and Tables

**Table 1:** Hydrogen-bond geometry (Å, °)

*D*—H⋯*A*	*D*—H	H⋯*A*	*D*⋯*A*	*D*—H⋯*A*
N1—H1*N*⋯Cl1	0.87 (2)	2.26 (2)	3.122 (1)	175 (2)
N1—H2*N*⋯Cl1^i^	0.94 (2)	2.11 (2)	3.040 (2)	169 (2)
C2—H2⋯Cl1	0.93	2.77	3.517 (1)	139
C5—H5⋯O1^ii^	0.93	2.55	3.451 (2)	164
C6—H6⋯Cl1^ii^	0.93	2.69	3.608 (2)	167
C7—H7*A*⋯O2^iii^	0.97	2.56	3.393 (2)	144

Symmetry codes: (i) 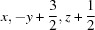; (ii) 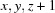; (iii) 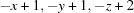.

**Fig. 1.**
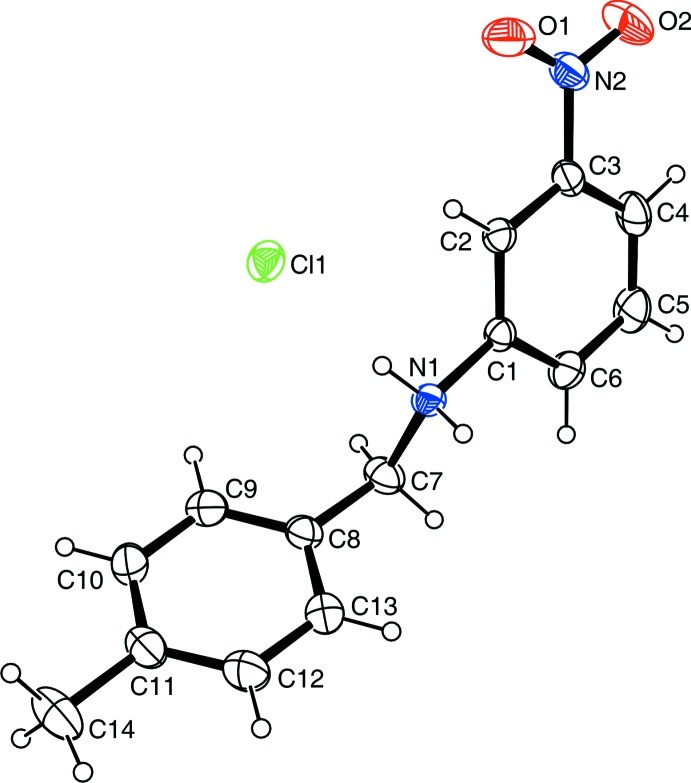
The molecular structure of the title compound with atom labelling. Displacement ellipsoids are drawn at the 30% probability level.

**Fig. 2.**
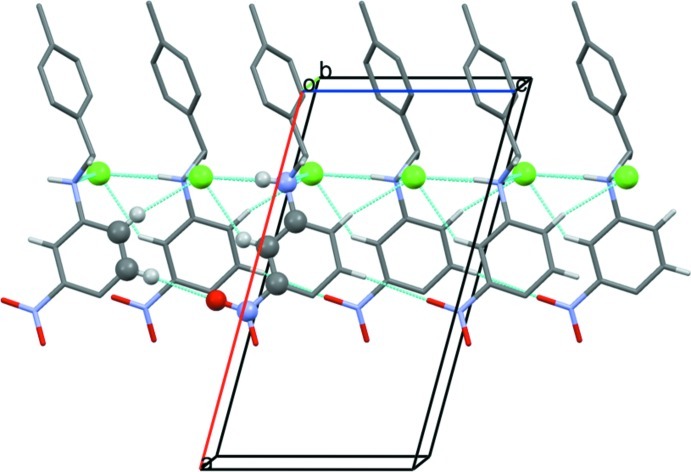
A partial view of the crystal packing of the title compound showing the infinite one-dimensional wavy chains running in direction [0 0 1] constructed via N—H···Cl hydrogen bonds and C—H···Cl and C—H···O interactions (dashed cyan lines) involving the chloride ions and the 3-nitrophenyl system. The atoms involved in the fused R12(6) and R23(10) ring motifs are shown as balls (see Table 1 for details).

**Fig. 3.**
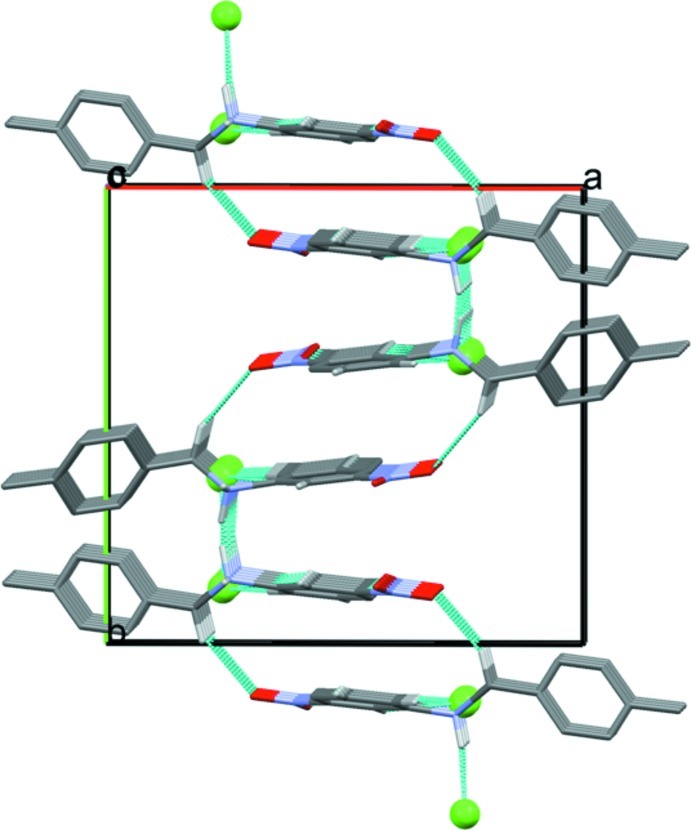
The crystal packing of the title compound viewed along the c axis. The N—H···Cl hydrogen bonds and the C—H···Cl and C—H···O interactions are shown as dashed cyan lines (see Table 1 for details).

## supplementary crystallographic information

### Comment

The title compound, *N*-(4-methylbenzyl)-3-nitroanilinium chloride, was prepared in a continuation of our laboratory work with high school students, in the scope of which we have recently reported the structure of the free base, *N*-(4-methylbenzyl)-3-nitroaniline (Đaković *et al.*, 2012).

As expected, the protonation of the nitrogen atom N1 in the title compound (Fig. 1), significantly influences its overall geometry in comparison with the recently reported structure of its free base mentioned above. In the latter the *N*-methyl-3-nitroaniline system is nearly ideally planar, while in the title cation only the 3-nitroaniline unit retains its almost ideally planar geometry (r.m.s. deviation of the atoms C1–C6/N1/N2/O1/O2 from their mean plane being 0.0117 Å, with atom N1 deviating from the plane by 0.041 (2) Å). Furthermore, atom C7 is pushed out of the plane by 0.840 (2) Å as a result of the changes in hybridization (*sp*^2^ to *sp*^3^) after protonation of atom N1. This feature is also reflected in the pronounced differences of the torsion angle C2—C1—N1—C7, that is 140.6 (2) ° for the title cation, and 0.8 (3) ° for the free base. Therefore, contrary to the bent conformation of the free base molecule, the cation of the title compound comprises two almost planar systems, 3-nitrophenyl (r.m.s. deviation = 0.0117 Å) and 4-methylphenyl (r.m.s. deviation = 0.238 Å), that are separated by the central C—N bond, with a dihedral angle of 61.36 (5) °.

In the crystal, neighbouring molecules are linked by N—H···Cl hydrogen bonds and C—H···Cl and C—H···O interactions generating one-dimensional chains running in the [0 0 1] direction (Fig. 2). Within these chains fused *R*^1^_2_(6) and *R*^2^_3_(10) ring motifs (Etter, 1990; Bernstein *et al.*, 1995) are formed via the C—H···Cl and C—H···O interactions. Parallel chains are further linked into a two-dimensional network *via* C—H···O interactions (Fig. 3).

### Experimental

The title compound was prepared by dissolving of *N*-(4-methylbenzyl)-3-nitroaniline, obtained as previously reported (Đaković *et al.*, 2012), in a methanolic solution of hydrogen chloride, 3% wt. Light-yellow block-like crystals, suitable for the X-ray diffraction analysis, were obtained by slow evaporation over 3–4 days.

### Refinement

The amine H atoms were located in a difference Fourier map and freely refined, giving N—H distances of 0.87 (2) and 0.94 (2) Å. The C-bound H atoms were placed in geometrically idealized positions and constrained to ride on their parent atoms: C—H = 0.93, 0.96 and 0.97 A for aromatic, methyl and methylene H atoms, respectively, with *U*_iso_(H) = k ×*U*_eq_(C), where k = 1.5 for methyl H atoms and = 1.2 for other H atoms.

### Crystal data

**Table:**

C_14_H_15_N_2_O_2_^+^·Cl^−^	*F*(000) = 584
*M**_r_* = 278.73	*D*_x_ = 1.321 Mg m^−^^3^
Monoclinic, *P*2_1_/*c*	Mo *K*α radiation, λ = 0.71073 Å
Hall symbol: -P 2ybc	Cell parameters from 3055 reflections
*a* = 14.2586 (7) Å	θ = 4.5–32.5°
*b* = 13.1416 (8) Å	µ = 0.27 mm^−^^1^
*c* = 7.7524 (3) Å	*T* = 296 K
β = 105.215 (5)°	Block, light-yellow
*V* = 1401.73 (13) Å^3^	0.56 × 0.46 × 0.14 mm
*Z* = 4	

### Data collection

**Table:**

Oxford Diffraction Xcalibur diffractometer with a Saphire-3 CCD detector	4075 independent reflections
Radiation source: Enhance (Mo) X-ray Source	2357 reflections with *I* > 2σ(*I*)
Graphite monochromator	*R*_int_ = 0.024
Detector resolution: 16.3426 pixels mm^-1^	θ_max_ = 30.0°, θ_min_ = 4.6°
CCD scans	*h* = −20→19
Absorption correction: multi-scan (*CrysAlis PRO*; Oxford Diffraction, 2009)	*k* = −18→9
*T*_min_ = 0.852, *T*_max_ = 0.964	*l* = −10→6
8156 measured reflections	

### Refinement

**Table:**

Refinement on *F*^2^	Secondary atom site location: difference Fourier map
Least-squares matrix: full	Hydrogen site location: inferred from neighbouring sites
*R*[*F*^2^ > 2σ(*F*^2^)] = 0.043	H atoms treated by a mixture of independent and constrained refinement
*wR*(*F*^2^) = 0.113	*w* = 1/[σ^2^(*F*_o_^2^) + (0.065*P*)^2^] where *P* = (*F*_o_^2^ + 2*F*_c_^2^)/3
*S* = 0.88	(Δ/σ)_max_ = 0.001
4075 reflections	Δρ_max_ = 0.31 e Å^−^^3^
181 parameters	Δρ_min_ = −0.20 e Å^−^^3^
0 restraints	Extinction correction: *SHELXL97* (Sheldrick, 2008), FC^*^=KFC[1+0.001XFC^2^Λ^3^/SIN(2Θ)]^-1/4^
Primary atom site location: structure-invariant direct methods	Extinction coefficient: 0.0056 (14)

### Special details

**Table:**

Geometry. Bond distances, angles *etc*. have been calculated using the rounded fractional coordinates. All su's are estimated from the variances of the (full) variance-covariance matrix. The cell e.s.d.'s are taken into account in the estimation of distances, angles and torsion angles
Refinement. Refinement on *F*^2^ for ALL reflections except those flagged by the user for potential systematic errors. Weighted *R*-factors *wR* and all goodnesses of fit *S* are based on *F*^2^, conventional *R*-factors *R* are based on *F*, with *F* set to zero for negative *F*^2^. The observed criterion of *F*^2^ > σ(*F*^2^) is used only for calculating -*R*-factor-obs *etc*. and is not relevant to the choice of reflections for refinement. *R*-factors based on *F*^2^ are statistically about twice as large as those based on *F*, and *R*-factors based on ALL data will be even larger.

### Fractional atomic coordinates and isotropic or equivalent isotropic displacement parameters (Å^2^)

**Table:**

	*x*	*y*	*z*	*U*_iso_*/*U*_eq_	
O1	0.57567 (10)	0.63507 (13)	0.82246 (19)	0.0791 (6)	
O2	0.68831 (9)	0.61154 (13)	1.0638 (2)	0.0867 (6)	
N1	0.26475 (8)	0.65004 (11)	1.00157 (16)	0.0369 (4)	
N2	0.60377 (10)	0.62354 (11)	0.9829 (2)	0.0541 (5)	
C1	0.36701 (10)	0.63347 (11)	1.09502 (17)	0.0347 (4)	
C2	0.43444 (10)	0.63446 (11)	0.99596 (18)	0.0372 (4)	
C3	0.53073 (10)	0.62306 (12)	1.08717 (19)	0.0404 (5)	
C4	0.56150 (12)	0.61009 (14)	1.2695 (2)	0.0543 (6)	
C5	0.49261 (13)	0.61086 (15)	1.3644 (2)	0.0588 (6)	
C6	0.39530 (12)	0.62219 (13)	1.27825 (19)	0.0478 (5)	
C7	0.19176 (11)	0.58625 (15)	1.0622 (2)	0.0502 (5)	
C8	0.09152 (11)	0.60310 (14)	0.94307 (19)	0.0445 (5)	
C9	0.05107 (12)	0.53434 (15)	0.8091 (2)	0.0528 (6)	
C10	−0.04264 (12)	0.54790 (15)	0.7043 (2)	0.0564 (6)	
C11	−0.09779 (12)	0.62937 (15)	0.7283 (2)	0.0541 (6)	
C12	−0.05627 (13)	0.69956 (17)	0.8588 (3)	0.0681 (7)	
C13	0.03705 (12)	0.68605 (16)	0.9664 (2)	0.0613 (7)	
C14	−0.20099 (14)	0.64248 (19)	0.6153 (3)	0.0808 (9)	
Cl1	0.24918 (3)	0.62691 (3)	0.59416 (4)	0.0470 (1)	
H1N	0.2565 (11)	0.6433 (12)	0.887 (2)	0.042 (4)*	
H2	0.41570	0.64250	0.87250	0.0450*	
H2N	0.2519 (14)	0.7188 (17)	1.020 (3)	0.073 (6)*	
H4	0.62700	0.60110	1.32670	0.0650*	
H5	0.51170	0.60370	1.48800	0.0710*	
H6	0.34910	0.62220	1.34350	0.0570*	
H7A	0.20890	0.51490	1.05980	0.0600*	
H7B	0.19290	0.60400	1.18420	0.0600*	
H9	0.08720	0.47860	0.78960	0.0630*	
H10	−0.06890	0.50060	0.61550	0.0680*	
H12	−0.09160	0.75670	0.87460	0.0820*	
H13	0.06320	0.73340	1.05520	0.0740*	
H14A	−0.22840	0.70300	0.65160	0.1210*	
H14B	−0.23900	0.58460	0.63070	0.1210*	
H14C	−0.20110	0.64820	0.49180	0.1210*	

### Atomic displacement parameters (Å^2^)

**Table:**

	*U*^11^	*U*^22^	*U*^33^	*U*^12^	*U*^13^	*U*^23^
O1	0.0592 (8)	0.1205 (14)	0.0652 (9)	0.0020 (8)	0.0300 (7)	−0.0021 (8)
O2	0.0341 (7)	0.1123 (14)	0.1114 (11)	0.0022 (8)	0.0153 (7)	−0.0063 (9)
N1	0.0342 (6)	0.0468 (9)	0.0297 (6)	0.0033 (6)	0.0083 (4)	0.0001 (5)
N2	0.0361 (7)	0.0534 (10)	0.0733 (10)	−0.0024 (7)	0.0154 (6)	−0.0096 (7)
C1	0.0341 (7)	0.0351 (8)	0.0327 (6)	0.0023 (6)	0.0047 (5)	−0.0007 (5)
C2	0.0364 (7)	0.0408 (9)	0.0327 (6)	0.0023 (7)	0.0061 (5)	0.0000 (6)
C3	0.0332 (7)	0.0370 (9)	0.0488 (8)	−0.0025 (7)	0.0067 (6)	−0.0047 (6)
C4	0.0399 (8)	0.0597 (12)	0.0518 (9)	0.0045 (8)	−0.0084 (7)	−0.0010 (8)
C5	0.0613 (11)	0.0724 (14)	0.0332 (7)	0.0026 (10)	−0.0044 (7)	0.0023 (7)
C6	0.0505 (8)	0.0602 (11)	0.0318 (7)	0.0014 (8)	0.0092 (6)	−0.0001 (7)
C7	0.0408 (8)	0.0648 (12)	0.0462 (8)	−0.0038 (8)	0.0138 (6)	0.0082 (7)
C8	0.0359 (7)	0.0576 (11)	0.0420 (8)	−0.0031 (7)	0.0137 (6)	0.0004 (7)
C9	0.0479 (9)	0.0566 (11)	0.0576 (9)	0.0014 (9)	0.0204 (7)	−0.0054 (8)
C10	0.0496 (10)	0.0692 (13)	0.0498 (9)	−0.0136 (10)	0.0121 (7)	−0.0114 (8)
C11	0.0398 (8)	0.0686 (13)	0.0520 (9)	−0.0047 (9)	0.0088 (7)	0.0064 (8)
C12	0.0465 (10)	0.0709 (15)	0.0858 (13)	0.0099 (10)	0.0153 (9)	−0.0137 (11)
C13	0.0468 (10)	0.0702 (14)	0.0646 (11)	−0.0019 (10)	0.0106 (8)	−0.0207 (9)
C14	0.0413 (10)	0.104 (2)	0.0879 (14)	−0.0025 (11)	0.0006 (9)	0.0170 (12)
Cl1	0.0535 (2)	0.0530 (3)	0.0344 (2)	−0.0059 (2)	0.0116 (1)	−0.0013 (2)

### Geometric parameters (Å, º)

**Table:**

O1—N2	1.212 (2)	C10—C11	1.370 (3)
O2—N2	1.214 (2)	C11—C12	1.382 (3)
N1—C1	1.464 (2)	C11—C14	1.512 (3)
N1—C7	1.505 (2)	C12—C13	1.384 (3)
N2—C3	1.477 (2)	C2—H2	0.9300
N1—H2N	0.94 (2)	C4—H4	0.9300
N1—H1N	0.87 (2)	C5—H5	0.9300
C1—C2	1.379 (2)	C6—H6	0.9300
C1—C6	1.379 (2)	C7—H7A	0.9700
C2—C3	1.378 (2)	C7—H7B	0.9700
C3—C4	1.376 (2)	C9—H9	0.9300
C4—C5	1.373 (2)	C10—H10	0.9300
C5—C6	1.381 (2)	C12—H12	0.9300
C7—C8	1.501 (2)	C13—H13	0.9300
C8—C13	1.378 (3)	C14—H14A	0.9600
C8—C9	1.383 (2)	C14—H14B	0.9600
C9—C10	1.381 (2)	C14—H14C	0.9600
			
Cl1···C2	3.5173 (14)	C6···H7B	2.8000
Cl1···C6^i^	3.6084 (17)	C6···H7A	3.0900
Cl1···N1	3.1224 (13)	C7···H6	2.7300
Cl1···N1^ii^	3.0398 (15)	C13···H2N	3.01 (2)
Cl1···C1^ii^	3.5683 (15)	C14···H12^ii^	3.0300
Cl1···H6^i^	2.6900	C14···H4^x^	2.9000
Cl1···H2	2.7700	H1N···Cl1	2.255 (15)
Cl1···H7B^i^	3.0800	H1N···H2	2.3000
Cl1···H1N	2.255 (15)	H2···O1	2.4100
Cl1···H10^iii^	3.1400	H2···H1N	2.3000
Cl1···H2N^ii^	2.11 (2)	H2···Cl1	2.7700
O1···C4^ii^	3.374 (3)	H2N···C13	3.01 (2)
O2···C7^iv^	3.393 (2)	H2N···Cl1^vi^	2.11 (2)
O1···H5^i^	2.5500	H4···O2	2.4200
O1···H2	2.4100	H4···H14C^xi^	2.5300
O2···H7A^iv^	2.5600	H4···C14^xi^	2.9000
O2···H4	2.4200	H5···O1^viii^	2.5500
O2···H14A^v^	2.7200	H5···C5^vii^	3.0500
N1···Cl1	3.1224 (13)	H6···C7	2.7300
N1···Cl1^vi^	3.0398 (15)	H6···H7B	2.2600
N2···C2^iv^	3.445 (2)	H6···Cl1^viii^	2.6900
C1···Cl1^vi^	3.5683 (15)	H7A···C6	3.0900
C2···C6^ii^	3.591 (2)	H7A···H9	2.3900
C2···N2^iv^	3.445 (2)	H7A···O2^iv^	2.5600
C2···C3^iv^	3.505 (2)	H7B···H6	2.2600
C2···Cl1	3.5173 (14)	H7B···Cl1^viii^	3.0800
C3···C3^iv^	3.526 (2)	H7B···C6	2.8000
C3···C2^iv^	3.505 (2)	H7B···H13	2.5200
C4···O1^vi^	3.374 (3)	H9···H7A	2.3900
C5···C5^vii^	3.567 (3)	H10···Cl1^iii^	3.1400
C6···C2^vi^	3.591 (2)	H12···H14C^vi^	2.3600
C6···Cl1^viii^	3.6084 (17)	H12···H14A	2.3500
C7···O2^iv^	3.393 (2)	H12···C14^vi^	3.0300
C7···C10^ix^	3.595 (2)	H13···H7B	2.5200
C8···C10^ix^	3.591 (2)	H14A···O2^xii^	2.7200
C10···C8^ix^	3.591 (2)	H14A···H12	2.3500
C10···C7^ix^	3.595 (2)	H14C···H4^x^	2.5300
C5···H5^vii^	3.0500	H14C···H12^ii^	2.3600
			
C1—N1—C7	116.3 (1)	C8—C13—C12	120.56 (17)
O1—N2—O2	124.2 (2)	C1—C2—H2	121.00
O1—N2—C3	118.1 (1)	C3—C2—H2	121.00
O2—N2—C3	117.6 (1)	C3—C4—H4	121.00
C1—N1—H2N	106.0 (13)	C5—C4—H4	121.00
C7—N1—H1N	110.1 (11)	C4—C5—H5	120.00
C7—N1—H2N	108.0 (13)	C6—C5—H5	120.00
H1N—N1—H2N	105.9 (17)	C1—C6—H6	120.00
C1—N1—H1N	109.9 (11)	C5—C6—H6	120.00
N1—C1—C2	118.2 (1)	N1—C7—H7A	110.00
N1—C1—C6	120.7 (1)	N1—C7—H7B	110.00
C2—C1—C6	121.09 (14)	C8—C7—H7A	110.00
C1—C2—C3	117.37 (13)	C8—C7—H7B	110.00
N2—C3—C4	118.8 (1)	H7A—C7—H7B	108.00
N2—C3—C2	118.0 (1)	C8—C9—H9	120.00
C2—C3—C4	123.15 (14)	C10—C9—H9	120.00
C3—C4—C5	117.99 (15)	C9—C10—H10	119.00
C4—C5—C6	120.72 (14)	C11—C10—H10	119.00
C1—C6—C5	119.67 (15)	C11—C12—H12	120.00
N1—C7—C8	110.5 (1)	C13—C12—H12	120.00
C7—C8—C13	120.98 (15)	C8—C13—H13	120.00
C9—C8—C13	118.50 (15)	C12—C13—H13	120.00
C7—C8—C9	120.52 (16)	C11—C14—H14A	109.00
C8—C9—C10	120.38 (17)	C11—C14—H14B	109.00
C9—C10—C11	121.48 (16)	C11—C14—H14C	109.00
C10—C11—C12	118.05 (16)	H14A—C14—H14B	109.00
C12—C11—C14	120.85 (18)	H14A—C14—H14C	109.00
C10—C11—C14	121.10 (17)	H14B—C14—H14C	109.00
C11—C12—C13	120.99 (19)		
			
C7—N1—C1—C2	140.58 (14)	C3—C4—C5—C6	1.3 (3)
C7—N1—C1—C6	−42.5 (2)	C4—C5—C6—C1	−0.4 (3)
C1—N1—C7—C8	−174.06 (13)	N1—C7—C8—C13	−81.38 (19)
O1—N2—C3—C2	0.8 (2)	N1—C7—C8—C9	99.82 (19)
O1—N2—C3—C4	−179.97 (17)	C7—C8—C9—C10	177.41 (16)
O2—N2—C3—C2	−178.65 (16)	C13—C8—C9—C10	−1.4 (3)
O2—N2—C3—C4	0.6 (2)	C7—C8—C13—C12	−178.34 (17)
C2—C1—C6—C5	−0.5 (2)	C9—C8—C13—C12	0.5 (3)
N1—C1—C2—C3	177.31 (13)	C8—C9—C10—C11	0.5 (3)
N1—C1—C6—C5	−177.32 (15)	C9—C10—C11—C14	−178.80 (17)
C6—C1—C2—C3	0.4 (2)	C9—C10—C11—C12	1.3 (3)
C1—C2—C3—C4	0.6 (2)	C14—C11—C12—C13	177.86 (18)
C1—C2—C3—N2	179.76 (13)	C10—C11—C12—C13	−2.3 (3)
N2—C3—C4—C5	179.37 (16)	C11—C12—C13—C8	1.4 (3)
C2—C3—C4—C5	−1.5 (3)		

Symmetry codes: (i) *x*, *y*, *z*−1; (ii) *x*, −*y*+3/2, *z*−1/2; (iii) −*x*, −*y*+1, −*z*+1; (iv) −*x*+1, −*y*+1, −*z*+2; (v) *x*+1, −*y*+3/2, *z*+1/2; (vi) *x*, −*y*+3/2, *z*+1/2; (vii) −*x*+1, −*y*+1, −*z*+3; (viii) *x*, *y*, *z*+1; (ix) −*x*, −*y*+1, −*z*+2; (x) *x*−1, *y*, *z*−1; (xi) *x*+1, *y*, *z*+1; (xii) *x*−1, −*y*+3/2, *z*−1/2.

### Hydrogen-bond geometry (Å, º)

**Table:**

*D*—H···*A*	*D*—H	H···*A*	*D*···*A*	*D*—H···*A*
N1—H1*N*···Cl1	0.87 (2)	2.26 (2)	3.122 (1)	175 (2)
N1—H2*N*···Cl1^vi^	0.94 (2)	2.11 (2)	3.040 (2)	169 (2)
C2—H2···Cl1	0.93	2.77	3.517 (1)	139
C5—H5···O1^viii^	0.93	2.55	3.451 (2)	164
C6—H6···Cl1^viii^	0.93	2.69	3.608 (2)	167
C7—H7*A*···O2^iv^	0.97	2.56	3.393 (2)	144

Symmetry codes: (iv) −*x*+1, −*y*+1, −*z*+2; (vi) *x*, −*y*+3/2, *z*+1/2; (viii) *x*, *y*, *z*+1.
